# LC-MS/MS-based phytochemical characterization and biological activities of the endemic species *Stachys milasensis*

**DOI:** 10.1039/d6ra04715g

**Published:** 2026-07-25

**Authors:** Busra Sisik, Cengiz Sarikurkcu

**Affiliations:** a Afyonkarahisar Health Sciences University, Faculty of Pharmacy Afyonkarahisar Türkiye sarikurkcu@gmail.com +90 507 191 25 24

## Abstract

*Stachys milasensis* Ö. Güner is a recently described endemic species of Türkiye for which phytochemical and biological data are currently unavailable. In this study, aqueous and methanolic extracts obtained from the aerial parts of *S. milasensis* were comprehensively evaluated for their phenolic composition, antioxidant capacity, and enzyme inhibitory activities. Total phenolic and flavonoid contents were determined spectrophotometrically, while individual phytochemicals were characterized using liquid chromatography-tandem mass spectrometry (LC-MS/MS). Antioxidant properties were assessed by DPPH, ferric reducing antioxidant power (FRAP), cupric reducing antioxidant capacity (CUPRAC), phosphomolybdenum, and metal-chelating assays, whereas enzyme inhibitory activities against cholinesterases, tyrosinase, α-amylase, and α-glucosidase were investigated. The methanolic extract exhibited higher total phenolic and flavonoid contents, reaching 77.69 mg gallic acid equivalents (GAE) per g extract and 71.53 mg rutin equivalents (RE) per g extract, respectively. LC-MS/MS analysis identified chlorogenic acid as the predominant constituent in both extracts, with concentrations of 10 613 and 11 160 µg g^−1^ extract in the aqueous and methanolic extracts, respectively. The methanolic extract also contained considerable amounts of verbascoside (730 µg g^−1^ extract), hyperoside (95.5 µg g^−1^ extract), and luteolin-7-glucoside (83.4 µg g^−1^ extract). Among the antioxidant assays, the methanolic extract demonstrated stronger DPPH radical scavenging (96.80 mg trolox equivalents (TE) per g extract), CUPRAC (297.89 mg TE per g extract), and FRAP (157.56 mg TE per g extract) activities. Furthermore, among the tested extracts, the methanolic extract exhibited the highest inhibitory activities against α-glucosidase (1009.48 mg acarbose equivalents (ACE) per g extract), α-amylase (247.54 mg ACE per g extract), and tyrosinase (67.79 mg kojic acid equivalents (KAE) per g extract). This study provides the first comprehensive phytochemical and biological characterization of *S. milasensis*, contributes the chemotaxonomic knowledge of the genus *Stachys*, and establishes a foundation for future comparative phytochemical studies.

## Abstract 2

*Stachys milasensis* Ö. Güner is a recently described endemic species of Türkiye for which phytochemical and biological data are currently unavailable.

## Graphical abstract

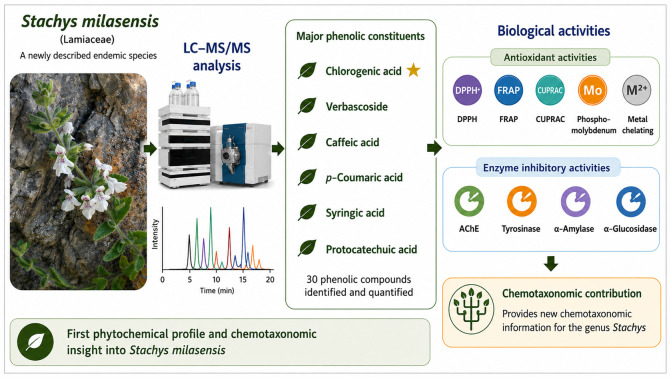

## Introduction

The family Lamiaceae comprises numerous medicinal and aromatic plant species recognized for their remarkable phytochemical diversity and broad spectrum of biological activities. Owing to their abundance of phenolic compounds, flavonoids, terpenoids, and essential oils, members of this family have been extensively utilized in traditional medicine and are increasingly investigated as valuable sources of natural bioactive compounds for pharmaceutical, nutraceutical, and food-related applications.^1^

Among the genera of Lamiaceae, *Stachys* is one of the largest and most diverse, consisting of more than 270 species distributed mainly throughout the Mediterranean Basin and Western Asia.^2^ Species of this genus have long been consumed as herbal infusions and decoctions and traditionally employed for the treatment of gastrointestinal disorders, inflammatory conditions, ulcers, respiratory ailments, and microbial infections. In parallel with their ethnopharmacological importance, numerous studies have demonstrated that *Stachys* species possess a wide range of biological properties, including antioxidant, antimicrobial, anti-inflammatory, anticholinesterase, antityrosinase, and antidiabetic activities.^1–4^ These biological effects have largely been attributed to the presence of phenolic acids, flavonoids, and phenylethanoid glycosides, which are recognized as major contributors to the bioactivity of the genus.

The growing interest in natural products has intensified efforts to identify plant-derived compounds capable of combating oxidative stress and modulating disease-related enzymes. Phenolic constituents are particularly attractive owing to their ability to scavenge reactive oxygen species, chelate transition metals, and inhibit oxidative processes associated with the pathogenesis of numerous chronic disorders.^5–7^ In addition to their antioxidant properties, these compounds have been reported to inhibit key enzymes involved in important health conditions. Inhibition of α-amylase and α-glucosidase is considered a promising strategy for controlling postprandial hyperglycemia in diabetes mellitus, whereas cholinesterase inhibitors are widely investigated for their potential role in the management of neurodegenerative disorders. Likewise, tyrosinase inhibitors have attracted considerable attention because of their applications in preventing enzymatic browning and treating hyperpigmentation-related disorders.^8–12^ Consequently, medicinal plants rich in phenolic constituents continue to represent an important reservoir of multifunctional bioactive molecules.

Within this context, endemic and insufficiently explored *Stachys* species constitute particularly valuable research targets because of their potential to yield novel phytochemicals and biological activities. *Stachys milasensis* Ö. Güner is a recently described endemic species of Türkiye that was formally introduced to the scientific literature in 2022 and classified within the section *Swainsoniana* of the genus *Stachys*.^13^ The species is morphologically distinguished from its closest relative, *S. swainsonii*, by several diagnostic characteristics and its geographically isolated distribution. The description of *S. milasensis* increased the number of *Stachys* taxa reported from Türkiye to 121, highlighting the remarkable diversity and endemism of the genus in the region.^13^ Despite its taxonomic significance and endemic status, no information is currently available regarding its phytochemical composition or biological properties. To the best of our knowledge, the phenolic profile, antioxidant capacity, and enzyme inhibitory potential of *S. milasensis* have not previously been investigated, representing a significant gap in the current literature.

Therefore, the present study was designed to provide the first comprehensive phytochemical and biological evaluation of *S. milasensis*. Beyond establishing the first phytochemical profile of this recently described endemic species, the study also sought to assess whether its major phenolic constituents could provide useful chemotaxonomic information for the genus *Stachys*. For this purpose, aqueous and methanolic extracts obtained from the aerial parts of the species were analyzed for their total phenolic and flavonoid contents, while their individual phytochemical constituents were characterized by LC-MS/MS analysis. Furthermore, antioxidant properties were assessed using multiple complementary *in vitro* assays, and enzyme inhibitory activities against cholinesterases, tyrosinase, α-amylase, and α-glucosidase were determined. By integrating detailed phytochemical characterization with biological activity assessment, this study provides the first comprehensive report on the bioactive potential of *S. milasensis* and establishes a scientific basis for future investigations of this newly described endemic species.

## Materials and methods

### Plant material

*Stachys milasensis* Ö. Güner aerial parts were collected during the flowering stage on 30 April 2023 from rocky habitats located along the Kayabükü–Çukurköy road in Milas, Muğla, Türkiye (37°27′57″ N, 27°40′44″ E; approximately 610 m above sea level). Botanical identification of the species was performed by Dr Olcay Ceylan (Department of Biology, Faculty of Science, Muğla Sıtkı Koçman University, Muğla, Türkiye).

The collected aerial parts were dried in a shaded environment with adequate air circulation at room temperature (25 ± 2 °C) for several weeks until a constant weight was attained. The dried plant material was subsequently ground using a laboratory mill to obtain a homogeneous powder suitable for extraction procedures.

### Preparation of extracts

The extraction procedure was performed using ultrasound-assisted extraction (UAE), an efficient technique widely employed for the recovery of phenolic compounds from plant matrices due to its ability to enhance mass transfer and improve extraction efficiency while reducing solvent consumption and processing time.^14^

Water and methanol were selected as extraction solvents because of their different polarities and their complementary capacities to recover a broad spectrum of hydrophilic phenolic constituents.^15^

Briefly, 10 g of powdered plant material was mixed with 200 mL of either distilled water or methanol and subjected to extraction in an ultrasonic bath operating at approximately 40 kHz and 250 W for 3 h. To minimize temperature increases caused by acoustic cavitation, the extraction vessels were externally cooled using an ice bath, and the extraction temperature was maintained at approximately 40 °C throughout the process. Temperature control during UAE is particularly important because excessive heating may promote degradation of thermolabile phenolic compounds and consequently influence extraction efficiency and extract composition.^14^

Following extraction, the mixtures were filtered through filter paper to remove plant residues. The methanolic extract was concentrated under reduced pressure at 40 °C using a rotary evaporator, whereas the aqueous extract was freeze-dried. The resulting dry extracts were stored at 4 °C until further analyses.

Extraction yields, calculated on a dry weight basis, were 16.21% and 13.40% for the aqueous and methanolic extracts, respectively.

### Determination of total phenolic and flavonoid contents

The total phenolic content (TPC) of the extracts was determined using the Folin–Ciocalteu colorimetric method, following the procedure reported by Sarikurkcu *et al.* (2020b) with minor modifications. Briefly, 0.25 mL of the extract solution was mixed with diluted Folin–Ciocalteu reagent (1 : 9, v/v), followed by the addition of 1% Na_2_CO_3_ solution. The reaction mixtures were incubated at room temperature for 2 h, and the absorbance was measured at 760 nm. Gallic acid was used as the calibration standard, and the results were expressed as mg gallic acid equivalents per g extract (mg GAE per g extract).^16,17^

The total flavonoid content (TFC) was evaluated using the aluminium chloride colorimetric assay. In this assay, 1.0 mL of extract solution was mixed with an equal volume of 2% AlCl_3_ solution. A blank solution was prepared using methanol instead of AlCl_3_. After incubation at room temperature for 10 min, the absorbance was recorded at 415 nm. Rutin was used for the calibration curve, and the results were expressed as mg rutin equivalents per g extract (mg RE per g extract).^18,19^

### LC-MS/MS-based phytochemical analysis

The phenolic composition of the aqueous and methanolic extracts was determined using a previously validated liquid chromatography-electrospray ionization-tandem mass spectrometry (LC-ESI-MS/MS) method.^20^ Quantitative analyses were performed on an Agilent Technologies 1260 Infinity liquid chromatography system coupled to an Agilent 6420 Triple Quadrupole mass spectrometer.

Chromatographic separation was achieved on a Poroshell 120 EC-C18 column (100 mm × 4.6 mm, 2.7 µm). The mobile phase consisted of water containing 0.1% formic acid (A) and methanol (B). The gradient elution program was as follows: 2% B at 0–3 min, 25% B at 6 min, 50% B at 10 min, 95% B at 14–17 min, and 2% B at 17.5 min. The column temperature was maintained at 25 °C, the flow rate was 0.4 mL min^−1^, and the injection volume was 2 µL.

The mass spectrometer was operated using an electrospray ionization source in both negative and positive multiple reaction monitoring (MRM) modes. The capillary voltage was set at −3.5/−4.0 kV in negative ion mode and 3.5/4.0 kV in positive ion mode. The drying gas temperature was 300 °C, the drying gas flow rate was 11 L min^−1^, and the nebulizer pressure was 40 psi. Target compounds were identified by comparing their retention times and precursor/product ion transitions with those of authentic reference standards. Quantitative results were expressed as µg g^−1^ extract.

Detailed LC-MS/MS operating parameters, MRM transitions, calibration data, limits of detection (LOD), and limits of quantification (LOQ) are provided in Tables S1 and S2 of the SI. Extract stock solutions were prepared at a concentration of 4 mg extract per mL. For chlorogenic acid analysis, both the aqueous and methanolic extract solutions were diluted 1 : 100, yielding average measured concentrations of 424.52 and 446.40 µg L^−1^, respectively, both of which were within the validated linear calibration range (1–500 µg L^−1^; *R*^2^ = 0.99948). The reported chlorogenic acid contents were obtained after applying the corresponding dilution factor. No analyte concentration was determined by extrapolation beyond the validated calibration range.

### Biological activity assays

#### Antioxidant activity assays

The antioxidant properties of the aqueous and methanolic extracts were evaluated using a battery of complementary *in vitro* assays based on different mechanisms of antioxidant action. Radical scavenging activity was determined using 2,2-diphenyl-1-picrylhydrazyl (DPPH˙) and 2,2′-azinobis-(3-ethylbenzothiazoline-6-sulfonic acid) (ABTS˙^+^) assays, whereas reducing power was assessed by cupric reducing antioxidant capacity (CUPRAC) and ferric reducing antioxidant power (FRAP) methods. In addition, total antioxidant capacity and metal ion chelating ability were determined using phosphomolybdenum and ferrous ion chelation assays, respectively.

All antioxidant assays were performed according to previously described procedures.^21–28^

For the phosphomolybdenum assay, the extracts were incubated with phosphomolybdenum reagent at 95 °C for 90 min, and absorbance was measured at 695 nm. DPPH radical scavenging activity was determined using a 0.004% methanolic DPPH solution after 30 min incubation in the dark at room temperature, and absorbance was recorded at 517 nm. ABTS radical cation scavenging activity was evaluated using pre-formed ABTS˙+ solution after 7 min incubation at room temperature, with absorbance measured at 734 nm.

The reducing capacity of the extracts was further assessed by CUPRAC and FRAP assays. For the CUPRAC assay, the reaction mixture containing CuCl_2_, neocuproine, and ammonium acetate buffer was incubated for 30 min at room temperature, and absorbance was measured at 450 nm. In the FRAP assay, the extracts were reacted with freshly prepared FRAP reagent for 30 min at room temperature and absorbance was recorded at 593 nm. Ferrous ion chelating activity was determined using the ferrozine method following 10 min incubation at room temperature, and absorbance was measured at 562 nm.

Trolox was used as the reference antioxidant for phosphomolybdenum, DPPH, ABTS, CUPRAC, and FRAP assays, whereas EDTA was used as the positive control for the metal chelating assay. The biological activities were expressed as mg Trolox equivalent (TE) per g extract or mg EDTA equivalent (EDTAE) per g extract, as appropriate. Complete experimental protocols, including reagent compositions, reaction volumes, incubation conditions, blank/control procedures, and calculation of biological activity parameters, are provided in the SI.

#### Enzyme inhibitory activity assays

The enzyme inhibitory activities of the extracts were evaluated against acetylcholinesterase (AChE), butyrylcholinesterase (BChE), tyrosinase, α-amylase, and α-glucosidase using previously validated spectrophotometric methods with minor modifications.^18,29^

AChE and BChE inhibitory activities were determined using Ellman's colorimetric method in a 96-well microplate. The reaction mixtures containing the extract, enzyme solution, and 5,5′-dithiobis-(2-nitrobenzoic acid) (DTNB) were pre-incubated for 15 min at 25 °C before initiating the reaction with acetylthiocholine iodide (ATCI) or butyrylthiocholine chloride (BTCl). Absorbance was measured at 405 nm after 10 min incubation, and appropriate blanks without enzyme were included to correct for background absorbance.

Tyrosinase inhibitory activity was determined using the modified dopachrome method with l-DOPA as the substrate. After pre-incubation of the extract with tyrosinase at 25 °C, the reaction was initiated by the addition of l-DOPA, and absorbance was recorded at 492 nm. Blank reactions prepared without enzyme were used for background correction.

The inhibitory activities against α-amylase and α-glucosidase were evaluated using the Caraway–Somogyi iodine/potassium iodide method and the *p*-nitrophenyl-α-d-glucopyranoside (pNPG) assay, respectively. The reactions were carried out in 96-well microplates under controlled incubation conditions, and absorbance was measured at 630 nm for α-amylase and 400 nm for α-glucosidase. Appropriate blank reactions without enzyme were included in all assays to eliminate interference caused by the extracts.

The inhibitory activities were expressed as mg galantamine equivalents per g extract (mg GALAE per g extract), mg kojic acid equivalents per g extract (mg KAE per g extract), and mg acarbose equivalents per g extract (mg ACE per g extract), respectively.

#### Expression of results and determination of EC_50_ and IC_50_ values

Antioxidant and enzyme inhibitory activities were expressed both as standard-equivalent values and as EC_50_/IC_50_ values to enable a more comprehensive comparison of extract potency.

For reducing power assays (CUPRAC and FRAP) and the phosphomolybdenum assay, EC_50_ (mg mL^−1^) was defined as the effective concentration corresponding to an absorbance value of 0.5. For DPPH, ABTS, and metal-chelating assays, IC_50_ (mg mL^−1^) represented the extract concentration required to achieve 50% radical scavenging activity or 50% inhibition of the ferrous ion-ferrozine complex formation. In enzyme inhibition assays, IC_50_ (mg mL^−1^) was defined as the extract concentration required to inhibit 50% of the corresponding enzyme activity. Lower EC_50_ and IC_50_ values indicate higher biological activity.

#### Statistical analysis

All experiments were carried out in triplicate, and the results are presented as mean ± standard deviation (SD). Statistical analyses were performed using IBM SPSS Statistics Version 22.0 (IBM Corp., Armonk, NY, USA).

Comparisons between two groups were conducted using Student's *t*-test. For datasets involving three or more groups, statistical significance was evaluated by one-way analysis of variance (ANOVA) followed by Tukey's honestly significant difference (HSD) post hoc test. Differences were considered statistically significant at *p* < 0.05.

For antioxidant and enzyme inhibitory assays, EC_50_ and IC_50_ values were calculated from concentration–response curves generated by linear regression analysis.

## Results and discussion

### Extraction yield, total phenolic and flavonoid contents

Ultrasound-assisted extraction afforded extraction yields of 16.21% and 13.40% for the aqueous and methanolic extracts of *S. milasensis*, respectively. Although the aqueous extract produced a higher extraction yield, the methanolic extract was considerably richer in phenolic constituents. This finding indicates that extraction yield alone does not necessarily reflect phytochemical richness, as water may extract larger quantities of highly polar non-phenolic constituents, including carbohydrates, proteins, and other hydrophilic metabolites, whereas methanol generally favors the recovery of phenolic compounds with diverse structural characteristics.^14,30^

The total phenolic and flavonoid contents of the extracts are presented in Table 1. Significant differences were observed between the two extracts (*p* < 0.05). The methanolic extract exhibited the highest total phenolic content (77.69 ± 4.60 mg GAE per g extract), whereas the aqueous extract contained 42.00 ± 0.14 mg GAE per g extract. A similar trend was observed for total flavonoid content, which increased from 19.82 ± 0.23 mg RE per g extract in the aqueous extract to 71.53 ± 1.60 mg RE per g extract in the methanolic extract. These results clearly demonstrate the superior capacity of methanol to recover phenolic constituents from *S. milasensis*.

**Table 1:** Extraction yield, total phenolic content and total flavonoid content of *S. milasensis* extracts^a^

Extract	Yield (%)	Total phenolic content (mg GAE per g extract)	Total flavonoid content (mg RE per g extract)
Aqueous	16.21 ± 0.85^a^	42.00 ± 0.14^b^	19.82 ± 0.23^b^
Methanolic	13.40 ± 0.56^b^	77.69 ± 4.60^a^	71.53 ± 1.60^a^

a Values are expressed as mean ± SD (*n* = 3). Different superscript letters within the same column indicate statistically significant differences between extracts according to Student's *t*-test (*p* < 0.05). GAE, gallic acid equivalent; RE, rutin equivalent.

The observed solvent-dependent differences may be attributed to the intermediate polarity of methanol, which facilitates the extraction of a broader range of phenolic acids, flavonoids, and phenylethanoid glycosides compared with water.^14^ Similar findings have been reported for several members of the genus *Stachys*. Methanolic extracts of *S. tmolea* exhibited markedly higher phenolic concentrations and antioxidant activities than corresponding aqueous extracts, emphasizing the crucial role of solvent polarity in determining phytochemical recovery.^30^ Likewise, enhanced accumulation of phenolic constituents in methanolic extracts has been reported for *S. cretica* subsp. *anatolica*,^31^*S. chasmosericea*,^32^ and *S. antalyensis*,^33^ where higher phenolic levels were generally accompanied by stronger biological activities.

When compared with other endemic *Stachys* taxa, the phenolic richness of *S. milasensis* appears noteworthy. The total phenolic content of the methanolic extract (77.69 mg GAE per g extract) exceeded those reported for *S. chasmosericea* (46.78 mg GAE per g extract) and *S. antalyensis* (58.29 mg GAE per g extract).^32,33^ Similarly, the flavonoid content of *S. milasensis* was substantially higher than the values reported for these species. Furthermore, the values obtained in the present study were generally higher than those reported for *S. cretica* subsp. *anatolica*^31^ and *S. tmolea*,^30^ suggesting that *S. milasensis* represents a particularly rich source of phenolic metabolites within the genus.

Phenolic compounds are widely recognized as major contributors to the biological properties of medicinal plants, including antioxidant, antidiabetic, anti-neurodegenerative, and enzyme inhibitory activities.^32–34^ Previous investigations on *S. tmolea*, *S. chasmosericea*, *S. antalyensis*, and *S. sparsipilosa* demonstrated strong associations between phenolic abundance and biological activity, particularly antioxidant capacity and enzyme inhibition.^30,32–34^ Therefore, the elevated total phenolic and flavonoid contents observed in the methanolic extract of *S. milasensis* suggest a greater potential for biological activity. This hypothesis was subsequently supported by LC-MS/MS profiling and bioactivity assays, in which the methanolic extract generally exhibited superior antioxidant and enzyme inhibitory properties. It should be noted that the quantitative values obtained by LC-MS/MS and the spectrophotometrically determined total phenolic content (TPC) are not directly comparable because the two methods measure different analytical parameters. LC-MS/MS provides absolute concentrations of individually identified phenolic compounds, whereas the Folin–Ciocalteu assay estimates the overall reducing capacity of the extract and expresses the results as gallic acid equivalents (GAE), reflecting the cumulative response of all reducing constituents rather than their actual mass concentrations. Consequently, the concentration of chlorogenic acid determined by LC-MS/MS represents only one component of the total phenolic profile, whereas the TPC values reflect the combined contribution of numerous phenolic constituents together with, to a lesser extent, other reducing compounds present in the extracts.^35,36^

### LC-MS/MS characterization of phenolic constituents

The phenolic composition of the aqueous and methanolic extracts of *S. milasensis* was characterized by LC-MS/MS analysis, and the quantitative results are summarized in Table 2. Among the 30 target compounds screened, 19 phenolic constituents were detected and quantified, whereas the remaining compounds were not detected in either extract. Significant differences were observed between the aqueous and methanolic extracts with respect to both qualitative and quantitative phytochemical composition, confirming the pronounced influence of solvent polarity on phenolic recovery.

**Table 2:** Phenolic compounds identified and quantified in aqueous and methanolic extracts of *S. milasensis* by LC-MS/MS analysis (µg g^−1^ extract)^a^

Compound	Aqueous extract	Methanolic extract
Chlorogenic acid	10 613 ± 21^b^	11 160 ± 35^a^
Verbascoside	25.6 ± 0.9^b^	730 ± 1^a^
Caffeic acid	582 ± 2^a^	89.0 ± 0.8^b^
*p*-Coumaric acid	152 ± 3^a^	19.6 ± 0.5^b^
Syringic acid	146 ± 2^a^	110 ± 8^b^
Protocatechuic acid	103 ± 1^a^	97.3 ± 0.3^b^
2,5-Dihydroxybenzoic acid	101 ± 3^a^	99.0 ± 0.4^a^
Hyperoside	21.2 ± 1.3^b^	95.5 ± 4.7^a^
Luteolin 7-glucoside	9.56 ± 0.71^b^	83.4 ± 0.6^a^
4-Hydroxybenzoic acid	50.2 ± 0.7^a^	24.5 ± 0.2^b^
3-Hydroxybenzoic acid	50.1 ± 2.0^a^	24.7 ± 0.1^b^
Kaempferol	45.0 ± 0.5^a^	20.0 ± 0.8^b^
Hesperidin	8.25 ± 0.42^b^	36.8 ± 0.9^a^
Apigenin	24.3 ± 1.0^a^	25.0 ± 1.6^a^
Ferulic acid	20.0 ± 1.1^a^	6.3 ± 0.1^b^
Vanillin	18.2 ± 0.1^a^	17.6 ± 0.8^a^
Rosmarinic acid	12.6 ± 0.1^a^	11.6 ± 1.9^a^
Luteolin	9.66 ± 0.48^a^	3.80 ± 0.04^b^
Apigenin 7-glucoside	3.93 ± 0.15^a^	4.44 ± 0.34^a^
(−)-Epicatechin	nd	nd
(+)-Catechin	nd	nd
2-Hydroxycinnamic acid	nd	nd
3,4-Dihydroxyphenylacetic acid	nd	nd
Eriodictyol	nd	nd
Gallic acid	nd	nd
Pinoresinol	nd	nd
Pyrocatechol	nd	nd
Quercetin	nd	nd
Sinapic acid	nd	nd
Taxifolin	nd	nd

a Values are expressed as mean ± SD (*n* = 3). Different superscript letters within the same row indicate statistically significant differences between extracts according to Student's *t*-test (*p* < 0.05). nd = not detected.

Chlorogenic acid was identified as the predominant compound in both extracts, reaching concentrations of 10 613 ± 21 µg g^−1^ extract in the aqueous extract and 11 160 ± 35 µg g^−1^ extract in the methanolic extract. In addition to chlorogenic acid, the methanolic extract contained considerable amounts of verbascoside (730 ± 1 µg g^−1^ extract), hyperoside (95.5 ± 4.7 µg g^−1^ extract), and luteolin 7-glucoside (83.4 ± 0.6 µg g^−1^ extract). By contrast, the aqueous extract was characterized by relatively higher concentrations of simple phenolic acids such as caffeic acid, *p*-coumaric acid, syringic acid, ferulic acid, and hydroxybenzoic acid derivatives. These findings indicate that methanol preferentially extracts glycosylated flavonoids and phenylethanoid glycosides, whereas water favors the recovery of smaller and more polar phenolic acids, a pattern previously reported for several *Stachys* taxa.^30–32^

The predominance of chlorogenic acid is consistent with previous phytochemical investigations conducted on members of the genus *Stachys*. Chlorogenic acid has been reported as one of the major constituents of *S. cretica* subsp. *anatolica*, *S. chasmosericea*, *S. antalyensis*, and *S. sparsipilosa*, suggesting that this hydroxycinnamic acid derivative may represent a characteristic chemotaxonomic marker within the genus.^30–34^ The consistently high abundance of chlorogenic acid among these species indicates a conserved phenolic biosynthetic pattern in *Stachys* and highlights its potential contribution to the biological properties commonly associated with the genus.

Another notable finding was the markedly higher concentration of verbascoside in the methanolic extract. Verbascoside is one of the most widely distributed phenylethanoid glycosides in *Stachys* species and has frequently been reported as a dominant constituent in several taxa. The presence of substantial amounts of verbascoside together with chlorogenic acid reinforces the phytochemical similarity of *S. milasensis* to other members of the genus. From a biological perspective, verbascoside is recognized as a potent antioxidant owing to its multiple hydroxyl groups and its ability to participate in electron-transfer and hydrogen atom transfer reactions. Previous studies have demonstrated that verbascoside-rich extracts generally exhibit enhanced radical scavenging and reducing power activities, suggesting that this compound may significantly contribute to the antioxidant profile of *S. milasensis*.^8,37^

The methanolic extract also contained appreciable amounts of hyperoside and luteolin 7-glucoside. Both compounds have frequently been detected in biologically active *Stachys* extracts and are considered contributors to antioxidant and enzyme inhibitory properties.^31–33^ The enrichment of these flavonoid derivatives in the methanolic extract is therefore consistent with the superior antioxidant and enzyme inhibitory activities observed in subsequent assays.

Interestingly, several simple phenolic acids, including caffeic acid, *p*-coumaric acid, ferulic acid, and syringic acid, were present at significantly higher concentrations in the aqueous extract. These phenolic acids are widely recognized for their antioxidant properties and are considered important contributors to the antioxidant potential of medicinal plants owing to their ability to donate electrons or hydrogen atoms and neutralize reactive oxygen species.^38,39^ Nevertheless, their lower overall abundance relative to chlorogenic acid and the flavonoid glycosides detected in the methanolic extract may partly explain the comparatively weaker performance of the aqueous extract in most antioxidant and enzyme inhibition assays. Similar composition–activity relationships have been reported for other *Stachys* species, where extracts enriched in chlorogenic acid, verbascoside, and flavonoid derivatives generally exhibited stronger antioxidant and enzyme inhibitory activities than extracts dominated by simpler phenolic constituents.^31,32,34,40^

Although verbascoside and glycosylated flavonoids are highly polar compounds, their preferential extraction by methanol does not necessarily contradict their high aqueous solubility. Extraction efficiency is determined not only by the intrinsic polarity of the analytes but also by several physicochemical factors, including solvent penetration into plant tissues, disruption of the plant matrix during ultrasound-assisted extraction, hydrogen-bonding interactions, solvent viscosity, and analyte–matrix interactions.^14,39^ Owing to its lower viscosity and greater ability to penetrate cellular structures, methanol often provides higher recoveries of phenolic glycosides and structurally complex phenolic constituents, despite their hydrophilic nature. Conversely, lower-molecular-weight phenolic acids, such as caffeic acid, may be extracted more efficiently into aqueous media. Therefore, the different distribution patterns observed for caffeic acid, verbascoside, and glycosylated flavonoids in the present study most likely reflect differences in extraction efficiency and compound–matrix interactions rather than aqueous solubility alone.

From a chemotaxonomic perspective, the predominance of chlorogenic acid together with the presence of verbascoside provides useful information on the phytochemical placement of *S. milasensis* within the genus *Stachys*. Chlorogenic acid has been reported as a major phenolic acid in several *Stachys* species and is not restricted to a single taxonomic section.^41,42^ Similarly, phenylethanoid glycosides, particularly acteoside/verbascoside, are widely recognized as characteristic constituents of the genus *Stachys*.^8,42^ Therefore, based on the currently available phytochemical evidence, the occurrence of these metabolites in *S. milasensis* is better interpreted as supporting its phytochemical affinity with the genus *Stachys* rather than indicating a chemotaxonomic marker specific to section *Swainsoniana*.

However, the available phytochemical information on taxa belonging to section *Swainsoniana* remains limited, and direct comparisons at the sectional level are currently insufficient. Previous metabolic studies have shown that related *Stachys* taxa may share common phenolic acids, phenylethanoid glycosides, and flavonoid derivatives, while some compounds may display section- or species-related distribution patterns.^41^ In this context, the chemical distinctiveness of *S. milasensis* appears to be associated not simply with the presence of chlorogenic acid and verbascoside, but with the strong predominance of chlorogenic acid in both extracts and its co-occurrence with verbascoside and flavonoid glycosides. Thus, the present LC-MS/MS profile contributes new chemotaxonomic information for this recently described endemic species and provides a reference point for future comparative studies within section *Swainsoniana*.

Overall, the LC-MS/MS results revealed that *S. milasensis* possesses a phenolic profile dominated by chlorogenic acid and supported by appreciable levels of verbascoside and flavonoid glycosides. The recurrent occurrence of chlorogenic acid as a major constituent across geographically distinct *Stachys* taxa suggests a conserved phenylpropanoid biosynthetic pathway within the genus and highlights its potential chemotaxonomic significance. Likewise, the co-occurrence of chlorogenic acid and verbascoside, both frequently reported as dominant metabolites in *Stachys* species, further reinforces the phytochemical affinity of *S. milasensis* with other members of the genus. The occurrence of these compounds may provide a plausible chemical basis for the antioxidant and enzyme inhibitory activities observed in the present study. To the best of our knowledge, this study provides the first comprehensive characterization of the phenolic composition of *S. milasensis*, thereby contributing valuable chemotaxonomic and pharmacological information for this endemic species.

### Antioxidant activity

The antioxidant activities of the aqueous and methanolic extracts of *Stachys milasensis* were evaluated using multiple *in vitro* assays based on distinct antioxidant mechanisms, including radical scavenging (DPPH and ABTS), reducing power (CUPRAC and FRAP), phosphomolybdenum total antioxidant capacity, and metal chelating activity. The results expressed as IC_50_/EC_50_ values and standard equivalent activities are presented in Table 3 and 4. Overall, the methanolic extract exhibited superior antioxidant performance in most assays, whereas the aqueous extract showed a greater metal chelating capacity.

**Table 3:** Antioxidant and enzyme inhibitory activities of *S. milasensis* extracts expressed as IC_50_/EC_50_ values (mg mL^−1^)^a^

Assay	Aqueous extract	Methanolic extract	Standard
Phosphomolybdenum (EC_50_)	0.96 ± 0.03^b^	0.97 ± 0.01^b^	0.39 ± 0.02^a^
DPPH (IC_50_)	3.87 ± 0.12^c^	2.07 ± 0.02^b^	0.20 ± 0.02^a^
ABTS (IC_50_)	2.43 ± 0.38^b^	1.98 ± 0.06^b^	0.21 ± 0.02^a^
CUPRAC (EC_50_)	1.35 ± 0.01^c^	0.57 ± 0.01^b^	0.17 ± 0.02^a^
FRAP (EC_50_)	0.52 ± 0.003^c^	0.29 ± 0.002^b^	0.043 ± 0.004^a^
Metal chelating (IC_50_)	1.69 ± 0.07^c^	2.54 ± 0.04^b^	0.019 ± 0.002^a^
AChE (IC_50_)	4.33 ± 0.29^c^	1.58 ± 0.03^b^	0.0028 ± 0.0002^a^
BChE (IC_50_)	5.73 ± 0.03^b^	na	0.0031 ± 0.0001^a^
Tyrosinase (IC_50_)	3.02 ± 0.03^c^	1.11 ± 0.002^b^	0.076 ± 0.004^a^
α-Amylase (IC_50_)	20.79 ± 0.86^c^	3.73 ± 0.03^b^	0.93 ± 0.01^a^
α-Glucosidase (IC_50_)	3.17 ± 0.07^c^	1.24 ± 0.01^b^	1.29 ± 0.06^a^

a Values are expressed as mean ± SD (*n* = 3). Different superscript letters within the same row indicate statistically significant differences according to one-way ANOVA followed by Tukey's post hoc test (*p* < 0.05). IC_50_ represents the concentration required to achieve 50% inhibition. EC_50_ represents the concentration producing an absorbance of 0.5 in the phosphomolybdenum, CUPRAC, and FRAP assays. Standard compounds: trolox (phosphomolybdenum, DPPH, ABTS, CUPRAC and FRAP), EDTA (metal chelating), galantamine (AChE and BChE), kojic acid (tyrosinase), and acarbose (α-amylase and α-glucosidase). na, no activity.

**Table 4:** Antioxidant and enzyme inhibitory activities of *S. milasensis* extracts expressed as standard equivalent activities^a^

Assay	Unit	Aqueous extract	Methanolic extract
Phosphomolybdenum	mg TE per g extract	417.62 ± 12.61^a^	413.80 ± 2.16^a^
DPPH	mg TE per g extract	51.67 ± 1.64^a^	96.80 ± 1.10^b^
ABTS	mg TE per g extract	83.27 ± 13.08^a^	100.98 ± 2.84^a^
CUPRAC	mg TE per g extract	125.93 ± 1.11^a^	297.89 ± 5.73^b^
FRAP	mg TE per g extract	86.22 ± 0.49^b^	157.56 ± 1.33^a^
Metal chelating	mg EDTAE per g extract	11.27 ± 0.46^a^	7.49 ± 0.13^b^
AChE	mg GALAE per g extract	0.65 ± 0.04^a^	1.78 ± 0.04^b^
BChE	mg GALAE per g extract	0.54 ± 0.003	na
Tyrosinase	mg KAE per g extract	24.83 ± 0.28^a^	67.79 ± 0.10^b^
α-Amylase	mg ACE per g extract	44.48 ± 1.83^b^	247.54 ± 1.86^a^
α-Glucosidase	mg ACE per g extract	393.87 ± 8.54^b^	1009.48 ± 4.91^a^

a Values are expressed as mean ± SD (*n* = 3). Different superscript letters within the same row indicate statistically significant differences between extracts according to Student's *t*-test (*p* < 0.05). TE, trolox equivalent; EDTAE, ethylenediaminetetraacetic acid equivalent; GALAE, galantamine equivalent; KAE, kojic acid equivalent; ACE, acarbose equivalent; na, no activity.

In the radical scavenging assays, the methanolic extract demonstrated significantly stronger activity than the aqueous extract. The DPPH IC_50_ value decreased from 3.87 ± 0.12 mg mL^−1^ for the aqueous extract to 2.07 ± 0.02 mg mL^−1^ for the methanolic extract, while the ABTS IC_50_ value decreased from 2.43 ± 0.38 to 1.98 ± 0.06 mg mL^−1^. Likewise, the DPPH activity expressed as Trolox equivalents almost doubled in the methanolic extract (96.80 ± 1.10 mg TE per g extract) compared with the aqueous extract (51.67 ± 1.64 mg TE per g extract). These findings are in good agreement with the significantly higher total phenolic and flavonoid contents of the methanolic extract as well as its enrichment in chlorogenic acid, verbascoside, hyperoside, and luteolin 7-glucoside. Phenolic compounds are widely recognized as effective radical scavengers owing to their ability to donate electrons or hydrogen atoms and stabilize reactive species.^38,39^ Similar relationships between phenolic abundance and radical scavenging activity have previously been reported for *S. cretica* subsp. *anatolica*, *S. chasmosericea*, *S. antalyensis*, and *S. sparsipilosa*, where phenolic-rich extracts consistently exhibited superior DPPH and ABTS scavenging capacities.^31–34^

A comparable trend was observed in the reducing power assays. The methanolic extract exhibited substantially lower EC_50_ values in both CUPRAC and FRAP assays than the aqueous extract, indicating a stronger electron-transfer capacity. This observation was further supported by the corresponding Trolox equivalent values, which reached 297.89 ± 5.73 mg TE per g extract for CUPRAC and 157.56 ± 1.33 mg TE per g extract for FRAP in the methanolic extract. In contrast, considerably lower values were obtained for the aqueous extract. The superior reducing power of the methanolic extract may be attributed to its high content of chlorogenic acid and verbascoside, both of which contain multiple hydroxyl groups capable of participating in electron-transfer reactions.^8,37^ Similar associations between phenolic richness and reducing power have been reported for several *Stachys* species, including *S. cretica* subsp. *anatolica*, *S. chasmosericea*, *S. antalyensis*, and *S. sparsipilosa*.^31–34^

Interestingly, the metal chelating assay exhibited a different pattern. The aqueous extract displayed a lower IC_50_ value (1.69 ± 0.07 mg mL^−1^) than the methanolic extract (2.54 ± 0.04 mg mL^−1^), indicating a greater ferrous ion chelating capacity. This result was also reflected in the EDTA equivalent values, which were higher for the aqueous extract (11.27 ± 0.46 mg EDTAE per g extract) than for the methanolic extract (7.49 ± 0.13 mg EDTAE per g extract). Unlike radical scavenging and reducing power assays, metal chelating activity is not always directly correlated with total phenolic content and may depend on the presence of other polar constituents capable of coordinating transition metal ions, including organic acids, peptides, proteins, and polysaccharides.^39,43^ Therefore, the stronger chelating activity of the aqueous extract may reflect the contribution of water-soluble constituents not fully represented in the LC-MS/MS profile.

In contrast to the other antioxidant assays, no statistically significant difference was observed between the extracts in the phosphomolybdenum assay. Both extracts exhibited comparable total antioxidant capacities, with values exceeding 400 mg TE per g extract and EC_50_ values close to 1 mg mL^−1^. The phosphomolybdenum assay evaluates the total antioxidant capacity of a sample through the reduction of Mo(vi) to Mo(v) by antioxidant compounds under acidic conditions and reflects the combined reducing capacity of all antioxidant constituents present in the extract, including both phenolic and non-phenolic compounds.^44^ Consequently, the comparable phosphomolybdenum activities observed for the two extracts may suggest that non-phenolic antioxidants in the aqueous extract partially compensate for its lower phenolic content. It should be acknowledged that the present study did not quantify non-phenolic reducing constituents, such as soluble sugars, proteins, organic acids, or other hydrophilic metabolites, which may contribute to the responses obtained in spectrophotometric assays, particularly the Folin–Ciocalteu and phosphomolybdenum methods.^35,36^ Therefore, the possible contribution of these constituents to the comparable phosphomolybdenum activities observed for the aqueous and methanolic extracts should be regarded as a plausible interpretation rather than a demonstrated conclusion. Further compositional analyses will be necessary to clarify the relative contributions of phenolic and non-phenolic constituents to the antioxidant capacity of *S. milasensis* extracts.

Overall, the antioxidant results clearly demonstrate that the methanolic extract possesses stronger radical scavenging and reducing properties than the aqueous extract. These findings are fully consistent with the higher total phenolic and flavonoid contents of the methanolic extract and its enrichment in chlorogenic acid, verbascoside, hyperoside, and luteolin 7-glucoside. Similar composition–activity relationships have consistently been reported for several *Stachys* species, including *S. cretica* subsp. *anatolica*, *S. chasmosericea*, *S. antalyensis*, and *S. sparsipilosa*.^31–34^ Collectively, these findings suggest that the antioxidant potential of *S. milasensis* is largely associated with its phenolic composition and provide a plausible phytochemical basis for the biological activities observed in the present study.

### Enzyme inhibitory activity

The enzyme inhibitory activities of the aqueous and methanolic extracts of *S. milasensis* were evaluated against acetylcholinesterase (AChE), butyrylcholinesterase (BChE), tyrosinase, α-amylase, and α-glucosidase. The results expressed as IC_50_ values and standard equivalent activities are presented in Table 3 and 4 Overall, the methanolic extract exhibited significantly stronger inhibitory activity than the aqueous extract against all tested enzymes except BChE.

The inhibition of cholinesterases is considered one of the principal therapeutic strategies for the symptomatic management of neurodegenerative disorders such as Alzheimer's disease, where reduced cholinergic neurotransmission is a characteristic pathological feature.^45,46^ The methanolic extract exhibited substantially stronger AChE inhibitory activity than the aqueous extract, with IC_50_ values of 1.58 ± 0.03 and 4.33 ± 0.29 mg mL^−1^, respectively. This observation was further supported by the corresponding galantamine equivalent values, which were considerably higher for the methanolic extract (1.78 ± 0.04 mg GALAE per g extract) than for the aqueous extract (0.65 ± 0.04 mg GALAE per g extract). In contrast, only weak BChE inhibition was observed for the aqueous extract, while the methanolic extract was inactive against this enzyme. Such selectivity toward AChE over BChE has occasionally been reported for phenolic-rich plant extracts and may be associated with structural differences between the active sites of the two enzymes, which can influence inhibitor binding affinity and substrate accessibility.^45,46^

Tyrosinase inhibition followed a similar pattern. The methanolic extract exhibited significantly stronger activity than the aqueous extract, with IC_50_ values of 1.11 ± 0.002 and 3.02 ± 0.03 mg mL^−1^, respectively. This observation was further supported by the corresponding kojic acid equivalent values, which were substantially higher for the methanolic extract. Tyrosinase is a copper-containing oxidase that plays a central role in melanogenesis and enzymatic browning processes; consequently, natural tyrosinase inhibitors have attracted considerable attention for cosmetic, pharmaceutical, and food-related applications.^47^ The stronger anti-tyrosinase activity of the methanolic extract may be associated with its higher abundance of chlorogenic acid, verbascoside, hyperoside, and luteolin derivatives, which have frequently been reported in biologically active *Stachys* extracts and are often linked to enhanced enzyme inhibitory properties.^31–33^

The most pronounced differences between the extracts were observed in the inhibition of carbohydrate-hydrolysing enzymes. The methanolic extract demonstrated substantially stronger α-amylase and α-glucosidase inhibitory activities than the aqueous extract, with IC_50_ values of 3.73 ± 0.03 and 1.24 ± 0.01 mg mL^−1^, respectively, compared with 20.79 ± 0.86 and 3.17 ± 0.07 mg mL^−1^ for the aqueous extract. Likewise, the corresponding acarbose equivalent values were considerably higher for the methanolic extract. Since α-amylase and α-glucosidase play central roles in the digestion of dietary carbohydrates and the subsequent release of glucose, inhibition of these enzymes is regarded as an effective strategy for controlling postprandial hyperglycaemia and managing type 2 diabetes.^48^ The superior inhibitory activity of the methanolic extract is consistent with its higher content of phenolic compounds, particularly chlorogenic acid, verbascoside, and flavonoid derivatives, which have frequently been associated with carbohydrate-hydrolysing enzyme inhibition. Similar relationships between phenolic-rich extracts and α-amylase/α-glucosidase inhibition have previously been reported for *S. antalyensis*, *S. chasmosericea*, and *S. sparsipilosa*.^32–34^

Overall, the superior enzyme inhibitory activities of the methanolic extract are consistent with its higher total phenolic and flavonoid contents and its enrichment in chlorogenic acid, verbascoside, hyperoside, and luteolin 7-glucoside. Previous studies on *Stachys* species have similarly demonstrated that extracts rich in phenolic acids, phenylethanoid glycosides, and flavonoids generally exhibit stronger cholinesterase, tyrosinase, and carbohydrate-hydrolysing enzyme inhibitory activities.^31–34^ These findings suggest that the phenolic constituents identified in *S. milasensis* may contribute to the observed enzyme inhibitory activities and support the view that this endemic species may serve as a source of bioactive phytochemicals for future phytochemical and pharmacological investigations. However, because the present study evaluated only crude extracts, the relative contribution of individual compounds could not be established. Further studies involving bioactivity-guided fractionation, isolation of active constituents, and evaluation of individual compounds will be required to confirm these composition–activity relationships.

## Conclusions

This study provides the first comprehensive phytochemical and biological evaluation of the endemic Turkish species *S. milasensis*. Ultrasound-assisted extraction using water and methanol yielded extracts with distinct chemical compositions and biological activities. The methanolic extract exhibited significantly higher total phenolic and flavonoid contents and generally demonstrated superior antioxidant and enzyme inhibitory activities compared with the aqueous extract.

LC-MS/MS analysis revealed chlorogenic acid as the predominant phenolic constituent of both extracts, while verbascoside, hyperoside, and luteolin 7-glucoside were also identified as important components of the phenolic profile. The enhanced antioxidant and enzyme inhibitory activities observed for the methanolic extract were consistent with its richer phenolic composition, suggesting that these metabolites may contribute substantially to the biological properties of *S. milasensis*. In contrast, the aqueous extract displayed a higher metal chelating capacity, indicating that different groups of phytochemicals may contribute to specific antioxidant mechanisms.

The findings presented herein contribute valuable phytochemical, chemotaxonomic, and pharmacological information for *S. milasensis*. In particular, the predominance of chlorogenic acid together with the occurrence of verbascoside and flavonoid glycosides represents a characteristic feature of the phytochemical profile of *S. milasensis* and provides new chemotaxonomic information that will facilitate future comparative studies within the genus *Stachys*. The demonstrated antioxidant, acetylcholinesterase (AChE) inhibitory, anti-tyrosinase, and carbohydrate-hydrolysing enzyme inhibitory activities suggest that *S. milasensis* may serve as a useful source of bioactive phytochemicals for future phytochemical and pharmacological investigations. However, further studies involving bioactivity-guided fractionation, isolation of active constituents, mechanistic investigations, and *in vivo* validation are required to confirm these biological effects.

## Author contributions

Busra Sisik: conceptualization, methodology, data curation, visualization, writing – original draft preparation. Cengiz Sarikurkcu: investigation, data curation, visualization, writing – original draft preparation, writing – review and editing.

## Conflicts of interest

All authors declare that they have no conflicts of interest.

## Supplementary Material

RA-OLF-D6RA04715G-s001

## Data Availability

The data supporting the findings of this study are available within the article and its supplementary information (SI) file. Additional data related to this work are available from the corresponding author upon reasonable request. Supplementary information: detailed LC-MS/MS operating parameters, MRM transitions, calibration data, limits of detection (LOD) and quantification (LOQ), together with detailed experimental protocols for the antioxidant and enzyme inhibitory assays. See DOI: https://doi.org/10.1039/d6ra04715g.

## References

[cit1] Grujic-Jovanovic S., Skaltsa H. D., Marin P., Sokovic M. (2004). Flavour Fragrance J..

[cit2] Sarikurkcu C., Ceylan O., Benabdallah A., Tepe B. (2021). S. Afr. J. Bot..

[cit3] Bahadori M. B., Kirkan B., Sarikurkcu C. (2019). Ind. Crops Prod..

[cit4] Bahadori M. B., Kirkan B., Sarikurkcu C., Ceylan O. (2019). Ind. Crops Prod..

[cit5] Ahmed I. A., Mikail M. A., bin Ibrahim M., bin Hazali N., Rasad M. S. B. A., Ghani R. A., Wahab R. A., Arief S. J., Yahya M. N. A. (2015). Food Chem..

[cit6] Carvalho M., Ferreira P. J., Mendes V. S., Silva R., Pereira J. A., Jerónimo C., Silva B. M. (2010). Food Chem. Toxicol..

[cit7] de Oliveira A. C., Valentim I. B., Silva C. A., Bechara E. J. H., Barros M. P. d., Mano C. M., Goulart M. O. F. (2009). Food Chem..

[cit8] Alipieva K., Korkina L., Orhan I. E., Georgiev M. I. (2014). Biotechnol. Adv..

[cit9] Nyambe-Silavwe H., Williamson G. (2018). Food Res. Int..

[cit10] Oboh G., Agunloye O. M., Akinyemi A. J., Ademiluyi A. O., Adefegha S. A. (2013). Neurochem. Res..

[cit11] Tadera K., Minami Y., Takamatsu K., Matsuoka T. (2006). J. Nutr. Sci. Vitaminol..

[cit12] Worsztynowicz P., Napierała M., Białas W., Grajek W., Olkowicz M. (2014). Process Biochem..

[cit13] Güner O. (2022). Phytotaxa.

[cit14] Azmir J., Zaidul I. S. M., Rahman M. M., Sharif K. M., Mohamed A., Sahena F., Jahurul M. H. A., Ghafoor K., Norulaini N. A. N., Omar A. K. M. (2013). J. Food Eng..

[cit15] Çelik S. E., Özyürek M., Güçlü K., Apak R. (2010). Talanta.

[cit16] Karakus Z. (2026). Int. J. Second. Metab..

[cit17] Sarikurkcu C., Uren M. C., Tepe B., Cengiz M., Kocak M. S. (2015). Ind. Crops Prod..

[cit18] Karakus Z. (2026). Molecules.

[cit19] Arumugam R., Kirkan B., Sarikurkcu C. (2019). S. Afr. J. Bot..

[cit20] Cittan M., Çelik A. (2018). J. Chromatogr. Sci..

[cit21] Apak R., Güçlü K., Özyürek M., Esin Karademir S., Erçaǧ E. (2006). Int. J. Food Sci. Nutr..

[cit22] Kocak M. S., Sarikurkcu C., Cengiz M., Kocak S., Uren M. C., Tepe B. (2016). Ind. Crops Prod..

[cit23] Tepe B., Sarikurkcu C., Berk S., Alim A., Akpulat H. A. (2011). Rec. Nat. Prod..

[cit24] Sarikurkcu C., Locatelli M., Mocan A., Zengin G., Kirkan B. (2020). Front. Pharmacol.

[cit25] Zengin G., Uren M. C., Kocak M. S., Gungor H., Locatelli M., Aktumsek A., Sarikurkcu C. (2017). Int. J. Med. Mushrooms.

[cit26] Karakus Z. (2026). ChemistrySelect.

[cit27] Sarikurkcu C., Uren M., Kocak M., Cengiz M., Tepe B. (2016). Food Sci. Biotechnol..

[cit28] Popović-Djordjević J., Cengiz M., Ozer M. S., Sarikurkcu C. (2019). Ind. Crops Prod..

[cit29] Sarikurkcu C., Andrade J. C., Ozer M. S., de Lima Silva J. M. F., Ceylan O., de Sousa E. O., Coutinho H. D. M. (2020). Food Chem..

[cit30] Elfalleh W., Kirkan B., Sarikurkcu C. (2019). Ind. Crops Prod..

[cit31] Carev I., Sarikurkcu C. (2021). LC-MS/MS Profiles and In Vitro Biological Activities of Extracts of an Endemic Species from Turkey: *Stachys cretica* ssp*. anatolica*. Plants.

[cit32] Kargin Solmaz F. O., Isitez N., Sarikurkcu C. (2025). Prep. Biochem. Biotechnol..

[cit33] Kargin Solmaz F. O. and Sarikurkcu C., Preparative Biochemistry & Biotechnology, In press, 2026 10.1080/10826068.2026.2620803 41582425

[cit34] Emir C., Buharalioglu G. Y., Ilhan R., Yildirim H., Çoban G., Emir A. (2026). Appl. Sci..

[cit35] Everette J. D., Bryant Q. M., Green A. M., Abbey Y. A., Wangila G. W., Walker R. B. (2010). J. Agric. Food Chem..

[cit36] Prior R. L., Wu X., Schaich K. (2005). J. Agric. Food Chem..

[cit37] Vertuani S., Beghelli E., Scalambra E., Malisardi G., Copetti S., Toso R. D., Baldisserotto A., Manfredini S. (2011). Molecules.

[cit38] Rice-Evans C. A., Miller N. J., Paganga G. (1996). Free Radic. Biol. Med..

[cit39] Dai J., Mumper R. J. (2010). Molecules.

[cit40] Karakus Z., Sarikurkcu C. (2026). Molecules.

[cit41] Tomou E.-M., Karioti A., Tsirogiannidis G., Krigas N., Skaltsa H. (2023). Agronomy.

[cit42] Pashova S., Karcheva-Bahchevanska D., Ivanov K., Ivanova S. (2024). Molecules.

[cit43] Apak R., Özyürek M., Güçlü K., Çapanoğlu E. (2016). J. Agric. Food Chem..

[cit44] Prieto P., Pineda M., Aguilar M. (1999). Anal. Biochem..

[cit45] Anand P., Singh B. (2013). Arch. Pharmacal Res..

[cit46] Lane R. M., Potkin S. G., Enz A. (2006). Int. J. Neuropsychopharmacol..

[cit47] Zaidi K. U., Ali A. S., Ali S. A., Naaz I. (2014). Biochem. Res. Int..

[cit48] Tundis R., Loizzo M. R., Menichini F. (2010). Mini-Rev. Med. Chem..

